# Protocol for production of homogeneous iPSC spheroids and microtissues using the suction technique

**DOI:** 10.1016/j.xpro.2025.103891

**Published:** 2025-06-12

**Authors:** Taijun Moriwaki, Hidenori Tani, Kotaro Haga, Shugo Tohyama

**Affiliations:** 1 Fujita Medical Innovation Center Tokyo, Fujita Health University, Ohta, Tokyo, Japan; 2 Kanagawa Institute of Industrial Science and Technology (KISTEC), Kawasaki, Kanagawa, Japan; 3 Keio University Regenerative Medicine Research Center, Kawasaki, Kanagawa, Japan; 4 Department of Cardiology, Keio University School of Medicine, Shinjuku, Tokyo, Japan

**Keywords:** Cell Biology, Stem Cells, Organoids, Tissue Engineering

## Abstract

Three-dimensional cultures mimic *in vivo* environments better than two-dimensional cultures and are often used in drug discovery research. Herein, we present a protocol for producing homogeneous induced pluripotent stem cell (iPSC) spheroids and microtissues using the suction technique. We describe steps for preparing the suction device, preparing and seeding cells, and suction sedimentation of cells. We then detail procedures for self-assembly and evaluation of spheroids.

For complete details on the use and execution of this protocol, please refer to Moriwaki et al.^1^

## Highlights

• Steps for preparing the suction device
• Instructions for producing homogeneous spheroids by suction technique
• Procedures to collect spheroids produced by suction technique

## Abstract (editor highlights)

Publisher’s note: Undertaking any experimental protocol requires adherence to local institutional guidelines for laboratory safety and ethics.

## Graphical abstract

**Figure:**
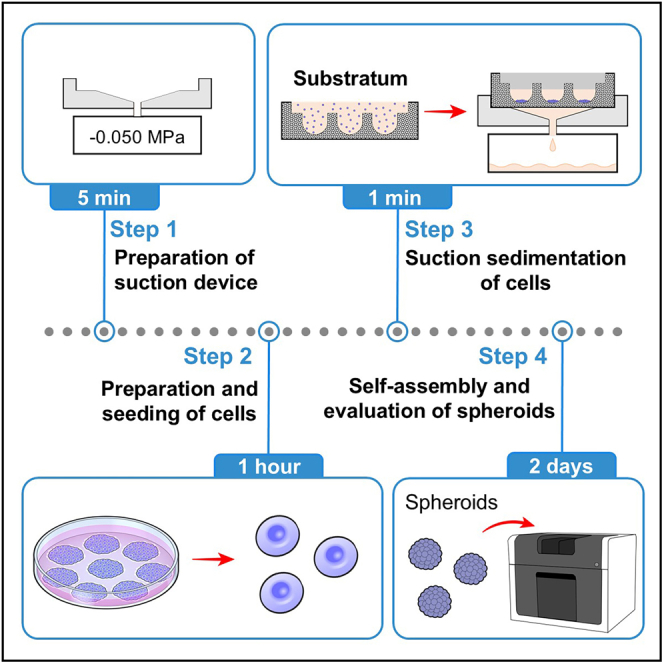

## Before you begin

In our previous research, we developed a “suction technique” for producing highly homogeneous spheroids/microtissues.^1^ This technique allows rapid cell aggregation by aspirating the medium from the cell suspension in the direction of the bottom of the well. To achieve this, we use a porous ceramic substratum made of TiO_2_. Ceramic substratum has been used to produce spheroids from various cells in other materials.^2^^,^^3^ The suction technique was shown to produce highly homogeneous spheroids/microtissues with higher efficiency compared with other spheroid production methods.

This protocol for spheroids production via the suction technique describes the specific procedure for using human induced pluripotent stem cells (hiPSC). This protocol was also used for hiPSC-derived cardiomyocytes (hiPSC-CM) for spheroids and microtissues production.

## Key resources table

**Table:** 

REAGENT or RESOURCE	SOURCE	IDENTIFIER
**Chemicals, peptides, and recombinant proteins**		

mTeSR1, cGMP	STEMCELL Technologies	Cat#85850
Y-27632	FUJIFILM Wako Pure Chemical	Cat#034-24024
Matrigel	Corning	Cat#354230
DMEM/F-12	Thermo Fisher Scientific	Cat#11320
D-PBS(−) [Ca^2+^ and Mg^2+^ free]	FUJIFILM Wako Pure Chemical	Cat#045-29795
TrypLE Select	Thermo Fisher Scientific	Cat#12563011
Bovine albumin fraction V	Thermo Fisher Scientific	Cat#15260037

**Experimental models: Cell lines**		

201B7	Provided by CiRA at Kyoto University	Takahashi et al.^4^

**Software and algorithms**		

GraphPad Prism 9	GraphPad	Cat#GPPEAC
BellCurve for Excel	Social Survey Research information	https://bellcurve.jp/ex/
Excel	Microsoft	N/A
Cell3iMager duos2	SCREEN	Cat#CC-8300

**Other**		

Substratum	CoorsTek	CEB-19-05-300 https://www.coorstek.com/en/contact/product-information/
Suction table	CoorsTek	CEB19-6 https://www.coorstek.com/en/contact/product-information/
O ring on the suction table	MISUMI	Cat#NPSW11
O ring VMQ-50 (SI50)	MORISEI KAKO	Cat#S-6
Three-way stopcock	TERUMO	Cat#TS-TR2K
Fitting adapter	Nordson MEDICAL	Cat#VFU306
Vacuum vessel	AS ONE	Cat#2-7875-01
Silicone tube	Masterflex	Cat#96400-16
MinicS-II	SHIN-EI Industries	Cat#23-6953-00
100 μm cell strainer	Falcon	Cat#352360

***Note:*** Substratum and suction table can be purchased or borrowed by contacting the following URL: (Outside of Japan) https://www.coorstek.com/en/contact/product-information/, (Japan) https://www.coorstek.com/jp/contact/product-and-solutions-inquiries/

CoorsTek corporate website URL: www.coorstek.com.

Contact email: (Outside of Japan) info@coorstek.com, (Japan) japaninfo@coorstek.com.

## Materials and equipment

**Table:** mTeSR1

Reagent	Final concentration	Amount
mTeSR1 Basal Medium	N/A	400 mL
mTeSR1 5x Supplement	1x	100 mL
**Total**	**N/A**	**500 mL**

Store at 4°C for up to 2 weeks.

**Table:** 0.1% BSA

Reagent	Final concentration	Amount
Bovine Albumin Fraction V	0.1%	100 μL
D-PBS(−)	N/A	7.4 mL
**Total**	**N/A**	**7.5 mL**

Use immediately after preparation.

**Table:** 10 mM Y-27632

Reagent	Final concentration	Amount
Y-27632	10 mM	25 mg
0.1% BSA	N/A	7.39 mL
**Total**	**N/A**	**7.39 mL**

Store at −20°C for up to 6 months.

## Step-by-step method details

### Thawing hiPSCs

**Timing: 1.5 h**

This step describes how to thaw frozen hiPSCs.

1. Add 10 mL of DMEM/F12 with 100 μL Matrigel to a 10 cm dish.

**CRITICAL:** Because Matrigel tends to solidify at room temperature, chill chips of micropipette and culture media at 4°C.

2. Incubate the dish at room temperature for 1 h.
3. At the same time, add Y-27632 to 25 mL of mTeSR1 (final concentration: 10 μM) and warm the medium to 37°C for 30 min.
4. Thaw a cryotube containing hiPSC in a water bath set at 37°C.
5. Transfer the hiPSCs from the cryotube to a 15 mL tube and add 10 mL of mTeSR1 with 10 μM Y-27632.
6. Centrifuge the hiPSCs at 300 × *g* for 3 min (±30 s).
7. Remove the supernatant and resuspend the hiPSCs in 10 mL of mTeSR1 with 10 μM Y-27632.
8. Remove the DMEM/F12 with Matrigel from the 10 cm dish and add 10 mL of the hiPSC suspension.
9. Incubate the cells at 37°C in a humidified 5% CO_2_ incubator.

### Medium change and passage of hiPSCs

**Timing: 2–8 weeks**

This step describes the method for culturing hiPSCs.

10. Replace the medium with mTeSR1 warmed to 37°C every 24–48 h until reaching >70% confluency.
11. Once >70% confluency is reached, prepare the Matrigel-coated 10 cm dish (Steps 1-2).
12. Remove the medium and rinse with 10 mL of D-PBS(−).
13. Add 2 mL of TrypLE Select enzyme (1 ×).
14. Incubate the cells at 37°C for 3 min (±10 s).
15. Detach the cells from the dish using a P1000 micropipette.
16. Add 10 mL of mTeSR1 with 10 μM Y-27632 and collect the cell suspension in a 15 mL tube.
17. Centrifuge the cells at 300 × *g* for 3 min (±30 s).
18. Remove the supernatant and resuspend in 2 mL of mTeSR1 with Y-27632.
19. Count the number of cells using Vi-CELL or any cell counting device.

***Note:*** We use Vi-CELL, an automated cell viability analyzer, to evaluate the cells before producing spheroids and use the cells with >90% viability.

20. Remove the medium with Matrigel from the 10 cm dish.
21. Seed 5 × 10^4^ – 2.0 × 10^5^ cells with 10 mL of mTeSR1 with 10 μM Y-27632 in the Matrigel-coated dish.

### Preparation of suction device

**Timing: 10 min**

This step describes the assembly method for the suction device.

**Figure 1:**
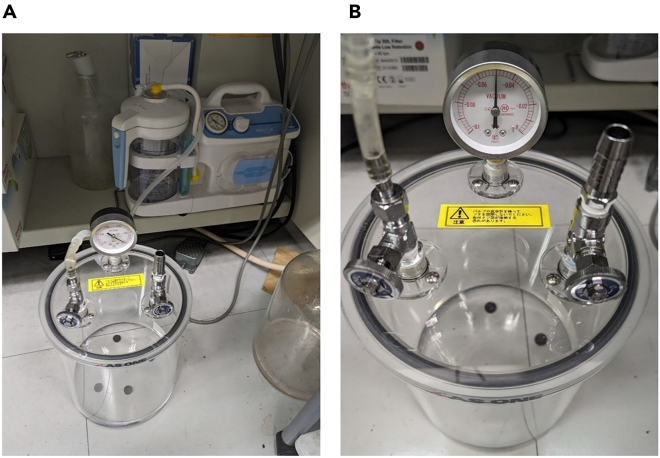
Removal of air from the vacuum vessel until −0.05 MPa is reached (A) Connect the vacuum vessel and pump (MinicS-II) to a silicon tube. (B) Pump the air out of the vacuum vessel until −0.05 MPa is reached.
23. Loosen the valve of the vacuum vessel.
24. Pump the air out of the vacuum vessel until the barometer on the vacuum vessel reads −0.05 MPa, as shown in Figure 1B (troubleshooting, problem 1).

***Note:*** We have confirmed that hiPSC spheroids and hiPSC-CS can be produced with high homogeneity at this suction force. However, we recommend that you consider the suction force when applying other cells to the suction technique.

**Figure 2:**
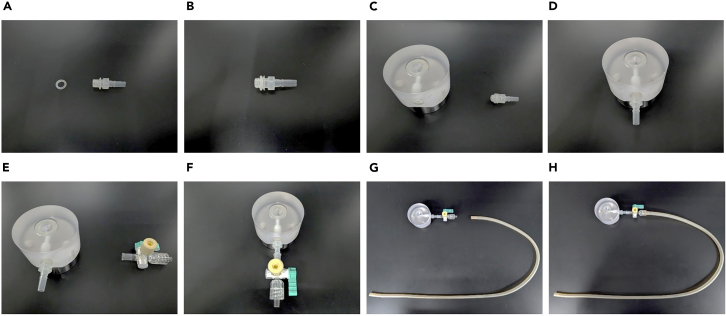
Assembly of the suction table (A and B) Fit the O-ring into the adapter. (C and D) Insert the fitting adapter into the suction table. (E and F) Connect the three-way stopcock to the suction table. (G and H) Connect the silicon tube to the three-way stopcock.

22. Connect the vacuum vessel and pump (Minic S-II) to a silicon tube, as shown in Figure 1A.

25. Tighten the valve of the vacuum vessel.
26. Fit the O-ring (MORISEI KAKO) into the adapter fitting, as shown in Figures 2A and 2B.

**CRITICAL:** The suction device should be assembled in a safety cabinet or clean bench to avoid contamination.

**CRITICAL:** Suction tables, O-rings, and adapter fitting must be sterilized by autoclaving before use.

27. Insert the fitting adapter into the suction table, as shown in Figures 2C and 2D.
28. Connect the three-way stopcock to a suction table, as shown in Figures 2E and 2F.
29. Connect the silicon tube to a three-way stopcock, as shown in Figures 2G and 2H.
30. Turn off the lever on the three-way stopcock, as shown in Figure 2H.
31. Connect the end of the silicon tube to the vacuum vessel.
32. Loosen the screw attached to the vacuum vessel.

### Preparation of substratum

**Timing: 3 min**

To stably aspirate the cell suspension on the substratum, the substratum should be soaked in the culture medium. This step describes the procedure for preparing the substratum.

**Figure 3:**
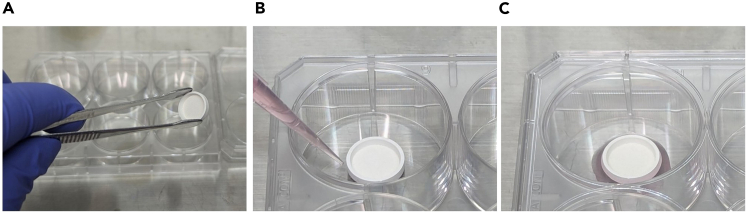
Preparation of substratum (A) Transfer the substratum to a 6 well plate with sterile tweezers. (B and C) Soak the substratum in 150 μL of culture medium.
34. Soak the substratum in 150 μL of mTeSR1 with 10 μM Y-27632, as shown in Figures 3B and 3C.

***Note:*** Drop the medium evenly around the substratum and allow it to soak from the outside of the substratum.

33. Transfer the substratum to a culture plate using sterile tweezers, as shown in Figure 3A.

35. Place the culture plate with the substratum in an incubator.

### Preparation of hiPSC suspension

**Timing: 30 min**

This step describes the procedure for preparing the hiPSC suspension to be added to the substratum.

36. Remove the medium and rinse with 10 mL D-PBS(−).
37. Add 2 mL of TrypLE Select enzyme (1 ×) and incubate 37°C for 3 min (±10sec).
38. Detach the cells from the dish using a P1000 micropipette.
39. Add 10 mL of mTeSR1 with 10 μM Y-27632 and collect the cell suspension in a 15 mL tube.
40. Centrifuge the cell suspension for 3 min at 300 × *g* (±30 s).
41. Remove the supernatant and resuspend in 2 mL of mTeSR1 with Y-27632.

***Alternatives:*** Frozen cells can be used to generate spheroids. We have used frozen cells to generate spheroids and microtissues from hiPSC-CM. However, we recommend that frozen cells be used within 3 months of freezing.

42. Prepare a cell suspension of 2.5 × 10^6^ cells/mL.
43. Count the number of cells.
44. Prepare the cell suspension in a 1.5 mL tube to a concentration of 2.5 × 10^6^ cells/mL.

**CRITICAL:** It is important to isolate the cells into single cells when they are detached. If the cells clump together after centrifugation, we recommend removing them using a cell strainer.

### Suction of hiPSC suspension

**Timing: 10 min**

This step describes the procedure of aspirating the medium from the cell suspension using a suction device.

45. Transfer the substratum onto a suction table using sterilized tweezers, as shown in Figure 4A.

**Figure 4:**
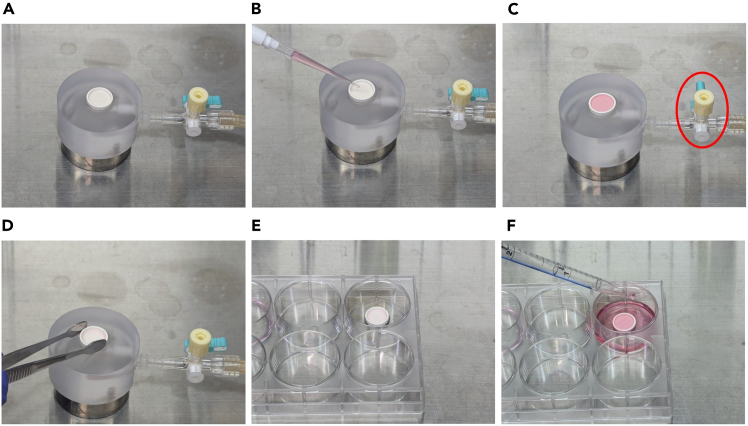
Suction of hiPSC suspension (A) Transfer the substratum onto the suction table using tweezers. (B) Soak the substratum in 150 μL of culture medium. (C) Turn on the lever on the three-way stopcock, as shown in the red circle. (D and E) Turn off the lever on the three-way stopcock once the center of the substratum is exposed. As soon as 70%–80% of the surface of the substratum is exposed, twist off the yellow cap and transfer the substratum to the culture dish with sterilized tweezers. (F) Add 3 mL of mTeSR1 with 10 μM Y-27632 from the corner of the culture dish.
46. Add 200 μL of cell suspension to the substratum, as shown in Figure 4B (troubleshooting, problem 2).
47. Turn on the lever of the three-way stopcock as shown in Figure 4C.
48. Once the center of the substratum is exposed, turn off the lever on the three-way stopcock (±2–3 s).
49. As soon as 70%–80% of the surface of the substratum is exposed, twist off the yellow cap and transfer the substratum to the culture dish using sterilized tweezers, as shown in Figures 4D and 4E.
50. Add 3 mL of mTeSR1 with 10 μM Y-27632 to the corner of the culture dish.
51. Gently place the culture dish in the incubator.

**CRITICAL:** Quick manipulation of steps 12–15 reduces cell damage.

***Note:*** Even when the lever on the three-way stopcock is turned off, the suction does not stop completely. A small amount of suction continues, and this is used to aspirate the medium until approximately 70%–80% of the surface of the substratum is exposed.

***Note:*** Multilayer microtissues composed of hiPSC-derived atrial and ventricular cardiomyocytes reported in our previous study were prepared as follows. First, hiPSC-derived atrial cardiomyocytes were aspirated, and when 70–80% of the substratum surface was exposed, an additional drop of hiPSC-derived ventricular cardiomyocyte suspension was added and aspirated.

***Note:*** We developed a substratum with 1069 microwells. In this protocol, 200 μL of 2.5 × 10^6^/mL cell suspension is added to the substratum, which means that each microwell contains approximately 468 cells.

### hiPSC spheroid culture

**Timing: 48 h**

This step describes the operation of the culture after aspiration is completed.

52. Remove the culture medium and add mTeSR1 without Y-27632 after 24 h of incubation.
53. Place the culture dish in the incubator and culture for 24 h.

### Recovery and evaluation of hiPSC spheroids

**Timing: 1 h**

This step describes the procedure for spheroid collection.

54. Remove the culture medium.
55. Flush 1 mL mTeSR1 into the microwells of the substratum using a P1000 micropipette to collect the spheroids.
56. Transfer the mTeSR1 to a well of a different noncoated 6 well plate.
57. Repeat steps 55–56 for approximately 10 times.
58. Acquire data on the spheroids collected using Cell3iMager duos2 or other suitable imaging instrument.

**CRITICAL:** Ensure that spheroids are evenly distributed on the dish before scanning. If the spheroids are crowded in one area of the dish, the distance between the spheroids will disappear and the boundaries between the spheroids will be indistinct. Particularly, when transporting dishes, spheroids often gather at the center of the dish. Therefore, it is important to verify before scanning that the spheroids do not gather at the center.

**CRITICAL:** Ensure that air bubbles are completely removed before scanning. If air bubbles are present on the surface of the culture medium in the dish, accurate recognition of the spheroids may not be possible.

## Expected outcomes

**Figure 5:**
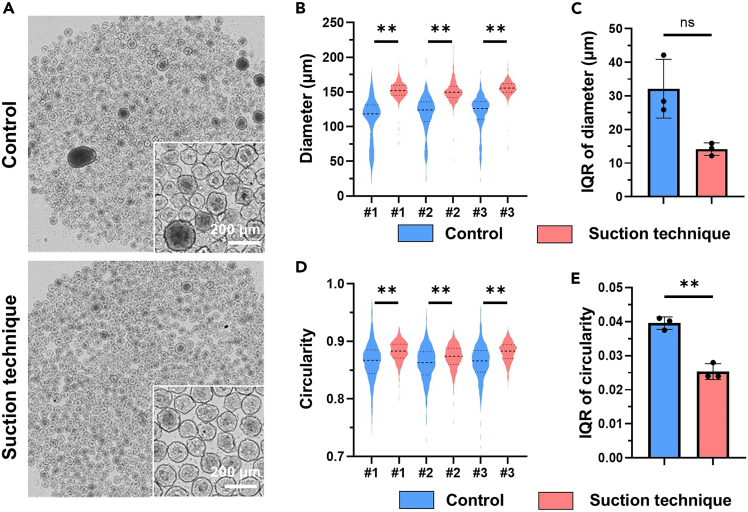
Evaluation of hiPSC spheroid homogeneity (A) Representative images of hiPSC spheroids using control technique and suction technique. (B) Violin plot of hiPSC spheroid diameter. #1-3 indicate the experiment number. Brunner–Munzel’s test, #1: n = 944 spheroids, #2: n = 986, #3: n = 1,049. (C) Interquartile range (IQR) of the hiPSC spheroid diameters. Welch’s test, n = 3. (D) Violin plot of hiPSC spheroid circularity. #1-3 indicate the number of experiments. Brunner-Munzel’s test, #1: n = 944 spheroids, #2: n = 986, #3: n = 1,049. (E) IQR of hiPSC spheroid circularity. Welch’s test, n = 3. Data are presented as the mean ± SD. ∗∗*p* < 0.01.

We have previously reported that the suction technique can produce spheroids/microtissues that are more homogeneous than those produced by spontaneous sedimentation (control technique).^1^ In this study, we produced spheroids using the hiPSC line 201B7. The diameters of the spheroids produced by the suction technique were significantly larger than those of the spheroids produced by the control technique (Figures 5A and 5B). Although the diameter deviation of the spheroids did not significantly differ between the two techniques, it tended to be smaller with the suction technique than with the control technique (Figure 5C). The circularity of the spheroids with suction technique was significantly higher than that with the control technique (Figure 5D). Finally, the deviation in circularity with the suction technique was significantly smaller than that with the control technique (Figure 5E).

In summary, the suction technique produced more homogeneous spheroids than the control technique and could be applied to other hiPSC lines and differentiated cells.^1^

## Quantification and statistical analysis

Statistical significance was determined through the utilization of Welch’s t-test or the Brunner-Munzel’s test. Welch’s t test was applied to assess the homogeneity of spheroid production. The Brunner–Munzel’s test was employed to evaluate parameters such as diameter, circularity of the spheroids. Because the data did not follow a normal distribution, especially for the control, and the diameters and circularities of the spheroids produced using the suction technique and the control technique are not homogeneity of variances, as shown by the violin plot, the Brunner–Munzel’s test was used to test for significant differences in the diameter and circularity of the spheroids. The Mann-Whitney U test is often used for testing non-normal distributions, but it should be avoided when testing data that does not show homogeneity of variances. The Brunner–Munzel’s test was performed using Bell Curve in Excel. Variations in the spheroid diameter and circularity were measured using the interquartile range (IQR) as an indicator. IQR rather than the standard deviation was used since some of the data did not follow a normal distribution.

## Limitations

We have previously reported differences in spheroid formation between different lots of cells, especially in the control technique.^1^ Therefore, the suction technique may also affect spheroid formation, owing to this lot-to-lot difference. We have shown that the suction technique can produce significantly more homogeneous hiPSC spheroids, hiPSC-CM spheroids and microtissues. However, the possibility of generating highly homogeneous spheroids and microtissues from other cell types remains unclear.

## Troubleshooting

### Problem 1

The air pressure inside the vacuum vessel does not decrease or returns immediately to its initial level even if the air pressure inside the vacuum vessel decreases.

### Potential solution

• Ensure that the valve on the vacuum vessel, which is not connected to the pump, is closed.
• Check the silicon tube connecting the vacuum vessel and pump for air leaks. If air leaks are found, replace the silicon tube.
• The O-ring fitted at the top of the vacuum vessel deteriorates over time and allows air to pass through. The O-ring must be replaced if there is a crack present.

### Problem 2

The medium cannot be aspirated.

### Potential solution

• The substratum may not be centered on the silicone ring of the suction table. If the substratum is misaligned, turn the lever off on the three-way stopcock and correct the position of the substratum using tweezers.
• The adapter fitting is a part of the suction device that is most prone to wear. Check for broken or cracked adapter fittings.
• The pressure inside the vacuum vessel often returns after it has been left for a few hours. Check the vacuum gauge to determine whether the pressure inside the vacuum vessel had returned.

## Resource availability

### Lead contact

Further information and requests for resources and reagents should be directed to and will be fulfilled by the lead contact, Shugo Tohyama (shugo.tohyama@fujita-hu.ac.jp).

### Technical contact

Technical questions on executing this protocol should be directed to and will be answered by the technical contact, Taijun Moriwaki (taijun.moriwaki@fujita-hu.ac.jp).

### Materials availability

This study did not generate new unique reagents. Details of the suction device are reported in the key resources table.

### Data and code availability

• All data reported in this paper will be shared by the lead contact upon request.
• This paper does not report the original code.
• Any additional information required to reanalyze the data reported in this paper is available from the lead contact upon request.

## Acknowledgments

We are grateful to Yu Yokoyama and Hiroko Watarai (CoorsTek GK) for their cooperation in substratum production. This work was supported by research grants from the Japan Agency for Medical Research and Development (AMED) (grant no. 25bm1223022 and 24bm1123010 to S.T.), KISTEC (to S.T.), and JSPS KAKENHI (grant no. 23K27683 to S.T.).

## Author contributions

T.M. performed and analyzed most of the experiments; the other authors contributed to specific experiments; T.M., H.T., K.H., and S.T. wrote the paper; S.T. acquired funding; and S.T. supervised the study.

## Declaration of interests

S.T. is an advisor at Heartseed Inc. S.T. owns equity in Heartseed Inc.
